# Ocular Radiation Exposure and Shielding Practices During Complex Fluoroscopic Guided Interventions: An Exploratory Multicenter Observational Study

**DOI:** 10.3390/tomography12070107

**Published:** 2026-07-20

**Authors:** Mathias Grau, Osama Eldergash, Sandeep Sunder Amin, Martin H. Maurer, Matteo Haupt, Vivek Chopda, Björn Poppe, Bernhard Schmuck, Arne Schwindt, Andreas Cöster, Torsten Schütz, Anika Wißmann, Rohit Philip Thomas, Christian Mathys

**Affiliations:** 1 Institute of Diagnostic and Interventional Radiology, Klinikum Oldenburg AöR, Carl von Ossietzky University Oldenburg, 26133 Oldenburg, Germanydrsandeepamin@yahoo.in (S.S.A.); maurer.martin@klinikum-oldenburg.de (M.H.M.); matteo.haupt@uni-oldenburg.de (M.H.); chopda.vivek@klinikum-oldenburg.de (V.C.); 2 Medizinisch-Technologische Schule für Radiologie, Bildungszentrum Klinikum Oldenburg AöR, Carl von Ozzietzky University Oldenburg, 26133 Oldenburg, Germany; 3 Department of Radiology, Faculty of Medicine, Misurata University, Misurata P.O. Box 2478, Libya; wesamdirgash@yahoo.com; 4 Department of Medical Radiation Physics, Pius-Hospital, Carl von Ossietzky University Oldenburg, 26133 Oldenburg, Germany; bjoern.poppe@uni-oldenburg.de; 5 Division of Radiology, Clinic for Vascular Medicine, Vascular Centre, Rotes Kreuz Krankenhaus, 28199 Bremen, Germany; schmuck.b@roteskrankenhaus.de; 6 Department of Vascular Surgery, St. Fransiskus Hospital, 48145 Munster, Germany; arne.schwindt@sfh-muenster.de; 7 Department of Vascular Surgery, Pius-Hospital, Carl von Ossietzky University Oldenburg, 26133 Oldenburg, Germany; andreas.coester@pius-hospital.de; 8 Department of Cardiology, Klinikum Oldenburg AöR, 26133 Oldenburg, Germany; schuetz.torsten@klinikum-oldenburg.de; 9 Institute of Diagnostic and Interventional Radiology, Ammerland Klinik GmbH, 26655 Westerstede, Germany; anika.wissmann@ammerland-klinik.de; 10 Institute of Radiology und Neuroradiology, Evangelisches Krankenhaus Oldenburg, Carl von Ossietzky University Oldenburg, 26122 Oldenburg, Germany; christian.mathys@uni-oldenburg.de; 11 Research Center Neurosensory Science, Carl von Ossietzky University Oldenburg, 26129 Oldenburg, Germany; 12 Department of Diagnostic and Interventional Radiology, University of Düsseldorf, 40225 Düsseldorf, Germany

**Keywords:** 2013/59/Euratom, ocular radiation dose, lead acrylic shield, radiation protection

## Abstract

**Background/Objectives:** Lead acrylic shields (LASs) enhance radiation protection, with variation of their usage among different specialties. This prospective multicentric observational study aimed to explore patterns of ocular radiation exposure and LAS usage among interventionalists from different disciplines and to assess behavioral dose changes in a longitudinally monitored subgroup. **Methods:** From July 2019 to July 2020, ocular doses (protected and unprotected) were measured in 15 interventionalists from four specialties (radiology, neuroradiology, cardiology and vascular surgery) across 2286 procedures using thermoluminescent dosimeters. Procedure type, operator position, total body dose, fluoroscopy time, dose area product, LAS usage and dose exceedances were documented. Ocular doses of five interventionalists, who participated in our previous study, allowed a longitudinal comparison of radiation doses and protective behavior. **Results:** Cumulative annual unprotected ocular doses ranged from 0 to 59 mSv, with five participants exceeding the annual limit of 20 mSv, whereas protected doses (0 to 18 mSv) remained within the limits. Procedural LAS usage was seen in 89% of radiologists and 82% of vascular surgeons. Participants with 100% LAS usage recorded minimal measurable ocular doses, whereas lower LAS compliance resulted in higher ocular doses. Longitudinal subgroup data analyses showed a decrease in ocular dose per procedure in all five participants, suggesting a potential learning effect, without statistical significance. **Conclusions:** Unprotected ocular doses exceeded the annual occupational eye lens dose limit in several participants, whereas protected doses remained within recommended limits in all participants. No specialty demonstrated universally lower ocular doses, while consistent LAS use proved highly effective in reducing the dose in all groups. Longitudinal findings suggest that structured dose monitoring and repeated training may support behavioral improvements in radiation protection over time.

## Simple Summary

Medical personnel performing complex endovascular interventions are exposed to high radiation doses, which may increase the cataract formation risk over time. In this multicentric multidisciplinary study, protected and unprotected eye lens doses were measured in 15 physicians during 2286 procedures. The present annual occupational dose limit was exceeded often, when no ceiling-mounted lead acrylic shield (LAS) was used consistently. No specialty demonstrated universally lower ocular doses for the same intervention type, while consistent LAS use proved highly effective in reducing the dose in all groups. Structured dose monitoring with repeated regular training may support behavioral improvements and increased radiation protection over time.

## 1. Introduction

Minimally invasive endovascular procedures are becoming increasingly popular nowadays and are very often associated with lesser mortality and morbidity and also play a vital role in shortening the patient stay in the hospital [1]. With increasing numbers of complex procedures in various disciplines like cardiology, interventional radiology and neuroradiology as well as vascular surgery, one of the main concerns shared by interventionalists across the specialties is the exposure to the ionizing radiation during such procedures [2,3]. Complex endovascular procedures are very often associated with high radiation doses consequent to relatively longer durations and proximity to the patient, resulting in high-scattered radiation [1,4].

Personal protective equipment such as lead aprons, thyroid shields and X-ray safety glasses are routinely used to reduce radiation exposure in clinical practice, where lead aprons and whole-body dosimeters are mandatory for personnel involved in such procedures [3,5]. Unprotected parts of the body including hands and the ocular lens that are exposed to radiation during procedures have been extensively discussed in the past years [6,7,8]. In complex procedures the ocular lens is especially susceptible to increased radiation dose and significant eye damage in the absence of proper radiation protective measures has been reported in the literature [8,9]. Accumulation of radiation doses over the years, even a dose lower than 2 Gy, could also lead to the risk of tissue reactions and cataract formation [10].

The annual permissible radiation ocular dose has been reduced by a factor of 7 from 150 to 20 mSv averaged over 5 y (100 mSv in 5 y) with no single yearly dose exceeding 50 mSv for occupational exposure in planned situations in the current European directive 2013/59/Euratom [11]. This amendment was consequent to the reported studies showing that a threshold dose is not required for cataract formation, and cataracts may be induced even at doses lower than 2 Gy. As opposed to the previous thresholds (0.5–2 Gy for a single exposure and 5 Gy for prolonged and fractional exposure), the threshold dose for radiation-induced cataract was thus revised to 0.5 Gy in ICRP Statement 2012 [12]. Consequently, X-ray safety glasses are nowadays increasingly used in clinical practice for the protection of eyes [3,13].

The unprotected ocular dose among the personnel involved in complex vascular procedures is reported by us to possibly cross the actual permissible ocular dose according to the new directive [14]. We then concluded that X-ray safety glasses would become mandatory in complex vascular procedures [14]. Another study from us in 2021 reported the efficacy of X-ray glasses in complex vascular procedures during 961 procedures, where the ocular dose was measured in front of and behind X-ray protective glasses worn by six interventional radiologists [15]. The authors reported that, although X-ray safety glasses provided measurable protection, the regulatory limit was exceeded in specific cases [15]. Similar studies showing that X-ray glasses do not protect from scattered radiation, particularly from lateral and overhead sources, have been reported in the literature [16]. To address the scattered radiation, ceiling-mounted lead acrylic shields (LASs) are widely employed in endovascular procedures in the angiography suite. These shields can substantially reduce operator dose, but their usage is known to vary between settings and individuals depending on procedural complexity, experience or even room configuration [2,17].

The present study, conducted between July 2019 and July 2020, represents a multicenter involvement including other endovascular disciplines in addition to interventional radiology, such as vascular surgery, cardiology and neuroradiology. The aim was to investigate whether ocular radiation exposure differs in endovascular procedures between the included endovascular disciplines and whether the frequency of LAS usage varies between specialties. In addition, the inclusion of five interventionalists, who have already participated in the earlier multicenter study, aimed at comparing the ocular radiation exposure over time and assessing the potential changes of radiation protective behavioral measures over time.

## 2. Material and Methods

### 2.1. Study Setting and Participants

This prospective, multicenter study was conducted from July 2019 to July 2020 in eight interventional departments across four disciplines including radiology, neuroradiology, vascular surgery, and cardiology. A total of 15 interventionalists participated, 11 of whom had between 10 and 20 years of professional experience, while 4 had 4 to 5 years. Participation was voluntary. Interventionalists from the participating departments were invited to take part in the study based on departmental availability and willingness to participate.

All participants wore standard personal protective equipment, including a lead apron (0.5 mm Pb equivalent), thyroid shield, and X-ray safety glasses. Two types of lead glasses were used: BR322 and BR126 (MAVIG GmbH, Munich, Germany), offering 0.75 mm lead equivalence anteriorly and 0.5 mm laterally. The specific type of lead glasses worn by each participant was not documented. However, both models provided identical frontal and lateral lead equivalence and are therefore assumed to offer comparable protection performance. In addition, a ceiling-mounted lead acrylic shield (LAS; 0.5 mm lead equivalence) was available in all units and was recommended for routine use to minimize scattered radiation exposure. Participants were instructed to use the LAS whenever possible and to document any situation where LAS usage was not possible.

The study was approved by the Medical Ethics Committee of the Carl von Ossietzky University before the commencement of the study. No additional radiation exposure was performed for research purposes. All recorded radiation exposure resulted exclusively from routine fluoroscopy-guided clinical procedures performed as part of standard patient care.

### 2.2. Procedural Characteristics

Routine procedures were typically performed by a single interventionist from the patient’s right side using a femoral approach in all vascular surgical, radiological and neuroradiological interventions. In the case of cardiological interventions, the predominant access was through the right radial artery, with a femoral approach used as bail out or in complex interventions. In complex procedures other than cardiology requiring additional time or bilateral access, two interventionists operated simultaneously from one or both sides. The type of procedure and the positioning of the interventionists relative to the patient were recorded for all cases.

All procedures were performed using angiography units equipped with automatic exposure control systems, with tube voltages ranging between 70 and 120 kV. Table-mounted lower body protection systems (MAVIG GmbH, Munich, Germany or Kenex (Electro-Medical) Ltd. (Harlow, Essex, UK)) were installed in all rooms. The field of view ranged from 11 to 48 cm. The radiological procedure spectrum included diagnostic angiographies, complex peripheral arterial revascularizations, stent graft implantations in the thoracic, thoracoabdominal, and abdominal aorta, embolizations, TIPS procedures, interventions for vascular malformations, and dialysis access procedures. Neurointerventional procedures included endovascular treatment of stroke, intracranial aneurysms, arteriovenous malformations, and cervical or intracranial stent implantations. The vascular surgical endovascular procedures included peripheral arterial revascularization, stent graft implantation in the thoracic, thoracoabdominal and abdominal aorta and embolizations. Femoral access was most common, with brachial or subclavian approaches used less frequently. The cardiological procedures included diagnostic coronary angiographies, stent angioplasty and complex interventions of the coronaries including rotation atherectomy, lithotripsies and laser angioplasties.

### 2.3. Radiation Dose Measurement

**Figure 1:**
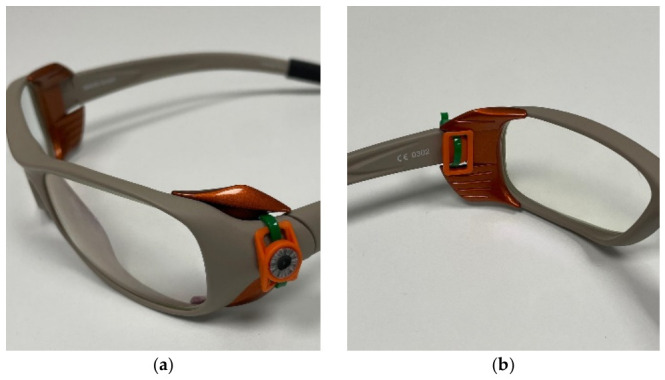
Thermoluminescent Dosimeter fixed to the outer (**a**) and inner (**b**) frames of the X-ray protective glasses to measure the unprotected and protected ocular doses.

Occupational exposure was monitored using personal dosimeters worn beneath the lead apron measuring the personal dose equivalent, *Hp* (10), which was used as an estimate of effective dose. The effective body dose was measured monthly using a film dosimeter placed beneath the lead apron. Protected and unprotected eye lens doses were measured using thermoluminescent dosimeters (TLDs) fixed to the inner and outer surfaces of the frame of the X-ray safety glasses, adjacent to the left eye—the side closest to the X-ray source as shown in Figure 1. The dosimeter attached to the outer surface of the glass frame was used to estimate the unprotected ocular dose, whereas the dosimeter attached to the inner surface represented the protected dose behind the radiation protection glasses. The eye lens dose was assessed using *Hp* (3) thermoluminescent dosimeters provided and evaluated by the certified Materials Testing Office NRW in Dortmund (Materialprüfungsamt, NRW, Dortmund, Germany) with a measurement range of 0.2–10 Sv. To eliminate background noise caused by natural ambient radiation during storage and transport, three TLDs were kept as controls. Their mean zero values of these TLDs were subtracted from the measured lens doses to isolate exposure due to procedural scattered radiation.

### 2.4. Data Collection and Variables

For each procedure, the dose area product (DAP), fluoroscopy time, LAS usage, and exceedance of diagnostic reference values were documented by the participating interventionalists.

### 2.5. Statistical Analysis

Accumulated radiation values and radiation dose per intervention were calculated using Microsoft Excel 2016 (Microsoft Corporation, Redmond, WA, USA). Statistical analyses and visualizations were performed in Python 3.11.12 using the packages SciPy (v1.15.3), NumPy (v2.0.2), plotnine, and matplotlib. The significance level was set at 0.05. To examine potential differences between the two measurement phases, Wilcoxon signed-rank tests were conducted for paired data. Additionally, linear regression analyses were used to assess associations between LAS usage and ocular radiation exposure.

## 3. Results

### 3.1. Overview of Procedures and Participants

This prospective, multicentric, multidisciplinary study involving eight interventional departments comprised a total of 2286 procedures performed by 15 interventionalists (nine interventional radiologists, five vascular surgeons, and one cardiologist) from July 2019 to July 2020. Each physician was exposed to varying radiation doses according to the number and complexity of the procedures, with the total number of the procedures per interventionalist ranging between 34 and 603 procedures. The *Hp* (10) values ranged from 0.0 to 1.4 mSv over the course of the study for all the procedures.

Five of the 15 interventionalists had also taken part in a previous single-center study conducted between July 2018 and July 2019, allowing a longitudinal comparison of radiation doses within the subset of individuals.

Detailed data on work experience, angiography systems, ocular radiation exposure (protected and unprotected), personal dose equivalent *Hp* (10), number of procedures, and percentage LAS usage are provided in Table 1.

### 3.2. Comparison of Ocular Dose Between Specialties

Because of the participation of only one cardiologist in the study, statistical group comparisons were limited to radiologists and vascular surgeons. The unprotected and protected ocular doses ranged between 0.0/0.0 mSv and 59/18 mSv, respectively. The annual limit for unprotected ocular exposure (20 mSv) was exceeded by five individuals: three radiologists and two vascular surgeons. In contrast, no exceedance of annual limits was observed for protected eye doses or whole-body doses.

Table 2 summarizes cumulative radiation exposure and fluoroscopic time per specialty and the type of intervention. Although average LAS usage was high in both groups (radiologists: 89%; vascular surgeons: 82%), there were marked differences in dose intensity across procedure categories. Notably, vascular surgeons performed more dose-intensive body interventions (average DAP per intervention: 393.64 Gy × cm^2^) than radiologists (119.69 Gy × cm^2^).

### 3.3. Longitudinal Comparison of Ocular Radiation Dose

Five interventionalists participated in the present study as well as a previous dose-monitoring study conducted between July 2018 and July 2019 (see Table 3). This overlap enabled a preliminary longitudinal comparison of ocular radiation exposure per procedure.

**Figure 2:**
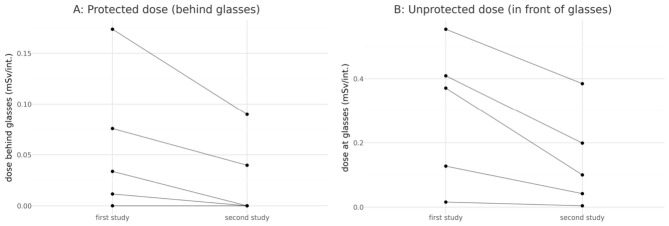
Change in eye lens dose per procedure between the initial and follow-up measurement phases: (**A**) Protected dose, measured behind the radiation protection glasses. (**B**) Unprotected dose, measured in front of the glasses. Paired data of five interventionists who participated in both the initial and follow-up measurement phases (conducted one year apart) show a consistent reduction in dose per intervention.

As shown in Figure 2A,B, all five individuals exhibited consistent dose reductions per intervention during the follow-up period. The mean protected dose decreased from 0.06 mSv (SD = 0.07) to 0.03 mSv (SD = 0.04), while the mean unprotected dose dropped from 0.30 mSv (SD = 0.22) to 0.15 mSv (SD = 0.15). Although these changes did not reach statistical significance, the Wilcoxon signed-rank tests yielded *p*-values close to conventional thresholds (protected: *p* = 0.068; unprotected: *p* = 0.063), indicating a trend-level effect.

### 3.4. Association Between LAS Usage and Eye Lens Dose

The effectiveness of the LAS in reducing ocular radiation exposure was evaluated by linear regression analyses. This included the examination of the association between LAS usage and both protected and unprotected eye lens doses.

For the protected dose, a significant negative correlation was found with increased LAS usage and was associated with lower radiation exposure (*r* = −0.79, *p* < 0.001, R^2^ = 0.62).

A similarly strong negative correlation emerged for the unprotected dose (*r* = −0.87, *p* < 0.001, R^2^ = 0.76).

In addition, a strong positive correlation was observed between the protected and unprotected doses (*r* = 0.93, *p* < 0.001, R^2^ = 0.87), indicating that participants with higher unprotected exposure also tend to accumulate higher protected doses.

**Figure 3:**
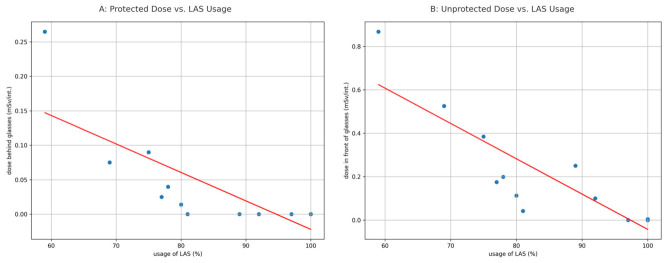
Relationship between lead acrylic shield (LAS) usage and ocular lens dose (**A**) Protected dose, measured behind the radiation protection glasses. (**B**) Unprotected dose, measured in front of the glasses. Linear regression analyses show a strong negative correlation between the frequency of LAS usage and radiation dose. More frequent LAS usage was associated with lower eye lens exposure in both protected and unprotected measurements (*r* = −0.78 and −0.87, respectively; *p* < 0.001).

These associations are illustrated in Figure 3, which displays scatter plots with regression lines for both protected (A) and unprotected (B) measurements. Higher LAS usage was consistently linked to lower eye lens dose, regardless of the measurement location.

## 4. Discussion

The frequency and complexity of fluoroscopic-guided interventional procedures along with the diagnostic radiological investigations, mainly computed tomography, contribute to the majority of human-made artificial radiation exposure to the population [2,3]. While the patients receive their radiation exposure from the investigations themselves, the medical staff and the personnel receive the same from the scattered radiation from the patient as well as the elements of the X-ray equipment. The radiation exposure could be very well associated with the occurrence of diseases like cataract formation, brain tumors, skin injuries as well as other inheritable defects [3,18,19,20,21,22]. In recent years, the importance of monitoring ocular radiation exposure during fluoroscopy-guided interventions has become increasingly recognized [3,8].

Our present multicenter prospective follow-up study included 15 interventionalists and, given the limited representation of cardiologists, statistical comparisons were limited to radiologists and vascular surgeons. Although only one cardiologist was included in the study, this interventionalist exhibited a substantially lower radiation exposure compared to the overall average of the interventionalists. This reduced dose might be attributed to the consistent use (100%) of the lead acrylic shield, despite the high number of performed interventions, which would typically be associated with increased exposure. With the limited number of cardiology and neuroradiology interventionalists, observations regarding these specialties should be interpreted with caution and are descriptive in nature.

The statistical comparison of the study demonstrates that not only interventional radiologists but also vascular surgeons may exceed the recommended dose limits for the eye lens, even when protective measures are in place. In both disciplines, ocular dose levels were closely associated with the frequency of use of the LAS, with both very low and very high exposure values observed depending on individual usage behavior (see Table 1 and Table 2). These findings confirm the results of an earlier, smaller study [14,15], which already highlighted the relevance of X-ray safety glasses and LASs in reducing radiation to the eye lens. In that previous study, relevant ocular dose levels were observed, and X-ray safety glasses alone were found to provide insufficient protection [15,16]. No consistent difference in radiation exposure between the two disciplines (radiology vs. vascular surgery) could be identified. High and low ocular doses were observed in both groups and across all types of interventions (see Table 2). Five participants (three radiologists and two vascular surgeons) exceeded the annual dose limit for the unprotected eye, while no exceedances were found for protected doses.

Despite prior training and the availability of shielding equipment, consistent use of the LAS was not universal in our study. Particularly in complex procedures, higher dose area product (DAP) values per intervention were associated with increased eye exposure, probably because of the missing possibility or difficulties in using the LAS in complex procedures. For instance, interventionalist no. 10 recorded a high average DAP of 274.91 Gy·cm^2^ and used the LAS only in 59% of procedures, resulting in unprotected and protected doses of 59 mSv and 18 mSv, respectively. In contrast, interventionalist no. 6, who performed a similar number of procedures with 100% LAS use, exhibited no measurable ocular dose (see Table 1). These findings are consistent with previous research demonstrating that consistent use of protective eye shielding, such as LASs or leaded eyewear, significantly reduces lens dose exposure [17].

While technical measures such as LASs and radioprotective eyewear are integral components of occupational radiation protection, the findings of this study suggest that their mere availability is insufficient to ensure optimal protection of the eye lens. A longitudinal comparison with the previous study (2018–2019) [14] was possible for five participants. As shown in Figure 2, all exhibited a consistent reduction in ocular dose per intervention during the follow-up period. The mean protected dose decreased from 0.06 mSv to 0.03 mSv, and the mean unprotected dose from 0.30 mSv to 0.15 mSv, although these changes did not reach statistical significance (Wilcoxon signed-rank tests: *p* = 0.068 for protected dose, *p* = 0.063 for unprotected dose). For all five participants of both studies except interventionalist no. 3, both the protected and unprotected doses, as well as average DAP and fluoroscopy time per intervention, decreased compared to the previous study (see Table 3). This consistent downward shift may reflect increased radiation awareness, e.g., caused by the involvement in the first study, improved compliance with protective measures such as LAS usage, or workflow adaptations over time. Due to the small sample size of the longitudinal comparison (*n* = 5), no definitive conclusions can be drawn, and these findings should also be interpreted with caution and may serve as a basis for further studies in the future.

The observed variability in ocular radiation exposure among interventionists, despite the presence of protective equipment, underlines the significant role of individual behaviors in influencing radiation safety outcomes. Specifically, the frequency and consistency of LAS usage were strongly correlated with lower ocular doses, highlighting the impact of personal adherence to safety protocols. This pattern aligns with previous research indicating that behavioral factors, including the regular application of protective measures, are critical determinants of radiation exposure levels [3,17]. Therefore, it could be hypothesized that the effectiveness of radiation protection strategies is not solely dependent on the technical attributes of protective devices but is also significantly influenced by the behavioral practices of healthcare professionals.

According to our findings, it is recommended that future radiation protection programs should emphasize the importance of the training of consistent and correct use of protective equipment, with the development of strategies to enhance the radiation protection and behavioral compliance among radiation medical personnel. Such an approach may lead to more effective reduction in ocular radiation exposure and overall improvement in occupational safety standards [3,18]. The hypothesis from the earlier study that greater experience inversely correlates with higher ocular dose was only partially supported. For instance, vascular surgeon no. 12 had 15 years of experience and a 0 mSv dose, whereas surgeon no. 10 had 20 years of experience and an unprotected dose of 59 mSv. This suggests that procedural type, personal shielding habits, and ergonomic factors may outweigh experience level as predictors of dose.

**Figure 4:**
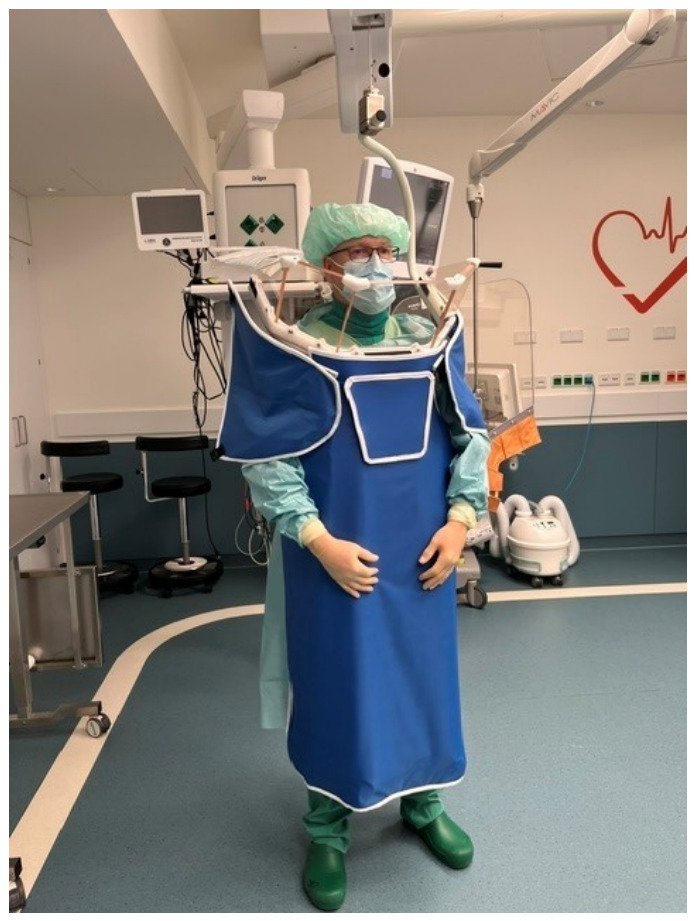
An example of zero-gravity shielding system with inbuilt ocular radiation protection and possible better mobility of the interventionalist.

Body and thoracic interventions emerged as the most dose-intensive categories (Table 2). These procedures are associated with larger examination volumes and closer proximity of the examiner, which probably increases the reception of scattered radiation. Notably, LAS usage was lowest in these cases (radiologists: 75%, vascular surgeons: 67%). This may be due to the restricted maneuverability of the LAS, leading even experienced operators to prioritize interventional efficiency over protection. The use of mobile zero-gravity shielding systems may offer a practical alternative and warrants further evaluation [17,23], Figure 4. As shown in Figure 3, linear regression analysis demonstrated a negative correlation between LAS usage and both protected and unprotected eye lens doses. Higher LAS usage was consistently associated with lower ocular exposure. However, these findings are based on aggregated participant-level data and should therefore be interpreted with caution, given the limited sample size [15,17].

**Figure 5:**
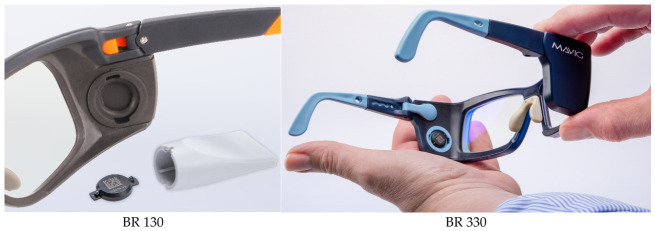
Radiation protection eyewear used for ocular dose monitoring with an integrated dosimeter attachment system.

Regular eye lens dose monitoring could become part of standard occupational safety protocols. In addition to X-ray safety glasses, consistent LAS use, side shielding, ergonomic optimization, and integration of real-time dosimetry are essential for effective dose reduction—particularly in complex abdominal and thoracic interventions [3,15,17]. Precise lens dose estimation could be best achieved using position-specific *Hp* (3) dosimeters such as MAVIG BR130 and BR330 with integrated dosimeters, whereas conventional whole-body or collar-mounted badges tend to under- or overestimate *Hp* (3) [24], Figure 5. The integration of a dosimeter into the protective glasses is considered particularly beneficial for procedures where higher radiation exposure to the eye lens has been observed.

This study has several limitations. At first, the sample size was relatively small, with only 15 interventionalists included overall. In addition, the participating specialties were unequally represented, which limits the ability to compare radiation exposure patterns between disciplines. LAS usage was documented by the participating interventionalists themselves, which may be subject to reporting bias. The exact positioning and adjustment of the ceiling-mounted lead acrylic shield and technical parameters such as tube angulation, frame rate, and magnification settings were not systematically recorded, although these factors may significantly influence the measured ocular doses. The statistical analyses were based on aggregate participant-level data. Ambient background radiation in the procedure rooms was not measured, which could have introduced additional variability, e.g., caused by heterogeneity of room design. Several recorded ocular dose values were reported as 0 mSv. It cannot be excluded that some of these values were below the measurable range of the dosimetry range rather than representing true absence of radiation exposure. Procedure types were not analyzed for each operator and the specific reasons for incomplete LAS use were not systematically documented. Although DAP and fluoroscopy time per procedure were reported, ocular dose was not additionally normalized to procedure number, DAP or fluoroscopy time for all operators. Thermoluminescent dosimeters were attached to the frame of the protective eyewear, providing an estimate of eye lens dose rather than a direct measurement at the lens itself. Similarly, the effective body dose was assessed using a single *Hp* (10) dosimeter worn beneath the lead apron and no additional collar or over the apron dosimeter was available. Therefore, these *Hp* (10) values may be interpreted rather as routing occupational data monitoring and not as a comprehensive scientific estimate of effective doses. Additionally, the primary focus of the study was not the effective dose but the directly measured protected and unprotected ocular dose. Future studies with larger cohorts and more detailed documentation of procedural parameters are warranted to validate and expand upon these findings.

## 5. Conclusions

This multicenter study demonstrates that consistent use of LAS is an important factor in reducing ocular radiation exposure during fluoroscopy-guided interventions. However, the availability of LASs and lead glasses does not guarantee full safety; rather, the frequency and consistency of their use with regular training are key determinants of exposure reduction. Infrequent LAS use correlated strongly with elevated ocular doses, especially in interventions with high scattered radiation such as abdominal and pelvic interventions. The findings highlight the need for radiation protection measurements with real-time dosimetry and structured feedback systems in the future for a safe interventional practice.

## Figures and Tables

**Table 1:** Participant Characteristics, Radiation Exposure, and LAS Usage: Overview of all 15 interventionalists included in the study, showing years of professional experience, angiography unit type used, cumulative unprotected and protected ocular doses (in mSv), cumulative personal dose equivalent *Hp* (10), number of interventions performed, and the proportion of procedures in which a ceiling-mounted lead acrylic shield (LAS) was used. Abbreviations: LAS = lead acrylic shield; mSv = millisievert; A–H = angiography systems: A: mono-planar, ceiling-mounted Axiom Artis (Siemens Healthineers, Forchheim, Germany), B: mono-planar, floor-mounted Artis Pheno (Siemens, Munich, Germany), C: bi-planar Artis dBA (Siemens), D: bi-planar Canon Infinix VF BP (Canon Medical Systems, Otawara, Japan), E: mono-planar, ceiling-mounted Artis Zee (Siemens Healthineers), F: mono-planar, ceiling-mounted Azurion 7 M20 with Flex Arm (Philips Healthcare, Amsterdam, The Netherlands).

No.	Specialty	Work Experience (Years)	Angiography Unit Type	Unprotected Dose (mSv)	Protected Dose (mSv)	Personal Dose Equivalent *Hp* (10) (mSv)	LAS Usage (%)	Number of Interventions
1	Radiologist	5	A/B	30	7	0.5	75	78
2	Radiologist	13	D	12	0	0.2	92	120
3	Radiologist	11	A/B	30	6	1.3	78	151
4	Radiologist	13	A/B	2	0	0.2	100	483
5	Radiologist	11	D	8	0	0.4	81	191
6	Radiologist	10	C	0.0	0	0	100	57
7	Radiologist	20	D	11	0	0.4	89	44
8	Radiologist	12	E	16	2	0.4	80	142
9	Neuroradiologist	5	A/B	21	3	0.9	77	120
10	Vascular Surgeon	20	E	59	18	1.4	59	68
11	Vascular Surgeon	20	E	42	6	0.9	69	80
12	Vascular Surgeon	15	F	0	0	0	100	44
13	Vascular Surgeon	5	F	0	0	0	100	71
14	Vascular Surgeon	2	F	0	0	0	100	34
15	Cardiologist	20	B	0	0	0	97	603

**Table 2:** Dose Metrics according to Specialty and Type of Intervention. Comparison of total cumulative annual and average dose area product (DAP), total cumulative and mean fluoroscopy time per intervention, number of procedures, and usage of a ceiling-mounted lead acrylic shield (LAS) among radiologists and vascular surgeons. Data are stratified by intervention type. Abbreviations: DAP = dose area product; LAS = lead acrylic shield; min= minutes; Gy = Gray.

	Total Cumulative Annual DAP for the Professional Group (Gyxcm^2^/Year)	Mean Cumulative Annual DAP per Interventionist (Gy·cm^2^/Operator/Year)	Mean DAP per Procedure (Gy·cm^2^/Procedure)	Total Cumulative Annual Fluoroscopy Time for the Professional Group (min/Year)	Mean Cumulative Annual Fluoroscopy Time per Interventionist (min/Operator/Year)	Mean Fluoroscopy Time per Procedure (min)	Number of Interventions	Interventions Performed with LAS	LAS Usage (%)
Cumulative Results: Radiologists versus Vascular Surgeons—All Interventions									
Radiologist	64,584.10	7176.01	46.60	22,092.47	2454.72	15.94	1386	1227	89
Vascular Surgeon	41,934.023	8386.80	141.19	5059.71	1011.94	17.04	297	244	82
Peripheral Interventions									
Radiologist	3506.46	389.60	7.08	5664.12	629.34	11.44	495	451	91
Vascular Surgeons	1796.19	359.23	14.60	975.33	195.06	7.9	123	109	89
Pelvic and Peripheral Interventions									
Radiologist	20,743.07	2304.78	39.36	7129.56	792.17	13.53	527	485	92
Vascular Surgeons	5041.21	1008.24	65.47	1271	254.22	16.50	77	67	87
Body Interventions									
Radiologist	30,384.44	3376.04	119.62	6339.79	704.42	24.96	254	191	75
Vascular Surgeons	35,034.213	7006.84	393.64	2760	552.00	31.01	89	60	67
Cervical and Intracranial Interventions									
Radiologist	9950.13	1105.57	90.46	2959	328.78	26.9	110	100	91
Vascular Surgeons	62.41	12.48	7.80	53.38	10.67	6.67	8	8	100

Note: Total cumulative annual DAP and total cumulative annual fluoroscopy time represent the summed values for each professional group and intervention category. Mean values per interventionist and per procedure were derived from these cumulative totals.

**Table 3:** Longitudinal Comparison of Ocular Dose and Procedural Parameters. Overview of five interventionalists who participated in both study phases (2018–2019 and 2019–2020), showing number of procedures, unprotected and protected eye lens doses, average dose area product (DAP), and average fluoroscopic time per intervention. Abbreviations: mSv = millisievert; DAP = dose area product; Gy·cm^2^ = Gray × square centimeter; min = minutes.

Interventionist	Number of Interventions		Unprotected Dose in Front of X-Ray Glasses (mSv)		Protected Dose Behind X-Ray Glasses (mSv)		Mean DAP/Intervention (Gy × cm^2^)		Mean Fluoroscopic Duration/Intervention, min	
	Study 1	Study 2	Study 1	Study 2	Study 1	Study 2	Study 1	Study 2	Study 1	Study 2
1 Rad.	121	78	67	30	21	7	90.1	71.23	28.48	22.76
2 Rad.	89	120	33	12	3	0	39.21	12.43	10.01	7.5
3 Rad.	66	151	27	30	5	6	92.37	75.13	23.31	20
4 Rad.	446	483	7	2	0	0	13.96	11.03	13.83	12.82
5 Rad.	173	191	22	8	2	0	41.12	35.6	9.01	7.2

## Data Availability

The data presented in this study are available on request from the corresponding author. The data are not publicly available because they contain individual occupational radiation exposure data and are subject to privacy and ethical restrictions.

## Abbreviations

LAS	Lead acrylic shield
TLD	Thermoluminescent dosimeter
DAP	Dose area product
mSv	Millisievert
Gy	Gray
SD	Standard deviation
ICRP	International Commission on Radiological Protection
